# Mitigation Potential of bio-fabricated selenium nanoparticles on arsenic induced stress in morpho-physiological growth of rice (*Oryza sativa* L.) seedlings

**DOI:** 10.1038/s41598-025-27108-4

**Published:** 2025-11-28

**Authors:** Jyotsna Setty, Pavan Singh, Sanjib Bal Samant, Mayank Kumar Yadav, Vijai Pandurangam

**Affiliations:** 1 Department of Plant Physiology, Institute of Agricultural Sciences, Banaras Hindu University, Varanasi, 221005 Uttar Pradesh India; 2 Faculty of Agricultural Sciences, GLA University, Mathura, 281406 Uttar Pradesh India; 3 Department of Mechanical and Industrial Engineering, Tallinn University of Technology, Tallinn, Estonia

**Keywords:** Selenium, Arsenic, ROS, Rice, Nanoparticles, Membrane stability index, Plant sciences, Environmental sciences, Nanoscience and technology

## Abstract

Arsenic (As) is a highly toxic metalloid that presents a major environmental hazard. Extensive contamination of agricultural soils by As is a global concern, necessitating the development of effective and cost-efficient strategies to mitigate its impact on food safety. Although selenium (Se) has been recognized for its antagonistic interactions with As, the potential of selenium nanoparticles (SeNPs) in mitigating As toxicity remains underexplored. In this study, biocompatible SeNPs were synthesized via a green approach using *Vitis vinifera* raisin extract and applied to rice (*Oryza sativa* L.) seedlings (HUR-105) through priming, co-application, and foliar spraying under As stress. Arsenic exposure significantly (p ≤ 0.01) impaired seedling growth by disturbing nutrient homeostasis, reducing chlorophyll biosynthesis, and weakening membrane stability. Application of SeNPs, particularly at 25 μM foliar concentration, substantially alleviated these effects by enhancing antioxidant enzyme activity, stimulating secondary metabolite production, and improving photosynthetic efficiency. Biochemical analyses revealed pronounced increases in chlorophyll (50%), carbohydrate (45%), soluble protein (48%), and free amino acid (44%) contents, alongside a 38% enhancement in membrane stability index. These findings indicate that SeNPs serves as an effective reactive oxygen species (ROS) quencher, mitigates As-induced oxidative damage by reinforcing redox homeostasis and metabolic activity. The study underscores the potential of SeNPs as a nanotechnological intervention to enhance stress resilience in rice, while highlighting the necessity of field-scale evaluations to establish dose optimization and long-term applicability under variable As conditions.

## Introduction

Heavy metals (HMs) are essential in trace amounts for optimal plant growth; however, their excessive accumulation disrupts normal metabolic and developmental processes in plants. Elevated concentrations of these metals significantly hinder plant development. Among them, As stands out as a highly toxic metalloid commonly found in the environment and is recognized as a Class 1 carcinogen by the World Health Organization^1^. Arsenic, predominantly of geogenic origin, is a major contaminant of groundwater- a critical resource for both drinking and irrigation purposes^2^. The global rise in groundwater contamination by As poses a serious threat to agricultural systems. Irrigation with As -contaminated water, along with various mobilization mechanisms, has led to increased As accumulation in soils, thereby facilitating As uptake by crop plants^3^. Notably, the translocation factor (TF) of As is greater in rice (0.8) than in wheat (0.1) and barley (0.2)^4,5^. This issue is particularly severe in the rice cultivation areas of Bangladesh, India, China, and Thailand, where rice is a dietary staple for a large portion of the population^6^. In India, the Ganga-Meghna-Brahmaputra plain constitutes a major rice-producing region, where As-contaminated groundwater poses a significant health risk to more than 500 million individuals^7^. Arsenic-contaminated rice has been specifically reported in the states of Chhattisgarh, Bihar, Jharkhand, Uttar Pradesh^8^, and West Bengal^9^, with further reports likely to emerge as groundwater contamination continues to be documented across several states^10^. Dietary exposure remains a critical concern, with daily As intake estimated at 560 µg for adults and 393 µg for children. Individuals with poor nutritional status are disproportionately affected by As toxicity compared with those with adequate nutrition^9^. Arsenic is taken up by plant roots and translocated to aerial parts, resulting in its accumulation and subsequent disruption of key physiological and metabolic functions^11^. Such exposure markedly impairs plant growth and development, ultimately reducing agricultural biomass yield. Many studies have identified oxidative stress and the depletion of photosynthetic pigments as the principal mechanisms underlying As-induced phytotoxicity^12^^,^^13^.

Plants have evolved multiple adaptive mechanisms to counteract As toxicity^14–16^. One such mechanism involves the competitive inhibition of toxic metal uptake by essential or less harmful elements, thereby restricting their absorption and assimilation. Se, in particular, has been shown to effectively reduce the uptake of HMs such as lead (Pb), cadmium (Cd), and As in species including cucumber, lettuce, and *Brassica*, respectively^17^^,^^18^^,^^19^^,^^20^^,^^21^.

Se is an essential micronutrient for humans and animals and is also beneficial to plants when it is present at appropriate concentrations^22^. Owing to the chemical similarity between Se and As, their biological interactions in plants can result in either antagonistic or synergistic effects on physiological functions^23^. Se mitigates heavy metal toxicity primarily by alleviating oxidative stress^24^, modulating light utilization^25^, promoting cellular repair, and regulating gene expression^26^. Additional mechanisms of Se-mediated detoxification include the downregulation of As transporter-related genes and the enhancement of plant defense systems and secondary metabolite synthesis under Se treatment^27^. Furthermore, Se supplementation has been shown to increase the levels of photosynthesis-related proteins, enzymes, and chlorophyll, thereby increasing photosynthetic efficiency and crop productivity^23^. Se also supports the expression of a variety of selenoproteins with key antioxidant and detoxification roles. As a potent antioxidant, Se reduces lipid peroxidation and increases glutathione peroxidase (GSH-Px) activity^24^. The application of Se has been associated with a reduction in As -induced oxidative damage, likely due to elevated levels of both enzymatic and non-enzymatic antioxidants Pandey & Gupta Pandey & Gupta^28^. The detoxification response in Se-treated plants includes increased production of metallothioneins (MTs), thiols, and glutathione S-transferase (GST). Supplementation with low concentrations of Se appears to enhance plant growth and fortify defense mechanisms against As toxicity^11^^,^^24^.

In the present study, a green synthesis approach was employed for the fabrication of SeNPs, utilizing plant extract-derived metabolites to meet the increasing demand for safe and sustainable nanoparticle synthesis methods. Compared with conventional chemical synthesis techniques, this biological method has demonstrated greater efficiency and environmental compatibility compared to conventional chemical synthesis techniques^29–32^. Although chemical methods remain widely used for nanoparticle production, many involve toxic reagents that pose environmental and health risks. In contrast, biological approaches employing microorganisms, enzymes, and plant-based materials have emerged as eco-friendly and viable alternatives to traditional physical and chemical synthesis routes^33^. In the present study, the bioreduction potential of raisin extract was employed for the synthesis of SeNPs. Raisins are rich in bioactive constituents such as sugars (~60%), flavonoids, phenolic compounds, minerals, iron, vitamins, potassium, and calcium^34^, which are likely to facilitate the reduction and stabilization of Se ions during nanoparticle formation. Compared with other Se forms, SeNPs exhibit distinct nanomaterial properties, including reduced toxicity, enhanced bioavailability, and superior biological activity^35,36^. These attributes position SeNPs as promising candidates for use as nano-fertilizers in agriculture, contributing to crop protection and improved nutritional quality^37–39^. However, research on the application of SeNPs for mitigating As toxicity in rice grains remains limited^40^. This study was conducted with the following specific objectives: (1) to examine the potential effects and underlying mechanisms by which varying concentrations of Se influence the morphological, physiological, and biochemical responses of rice under As stress,and (2) to evaluate the impact of different Se application methods on these morpho-physiological and biochemical parameters under As stress conditions. The central hypothesis of this study is that Se functions as an effective ROS quencher, thereby mitigating As toxicity by reducing As accumulation and alleviating As -induced oxidative stress.

While numerous studies have investigated the influence of Se, in both its bulk and salt forms, on plant growth, this research represents the first systematic assessment of the effects and mechanisms of SeNPs on rice under As stress. The outcomes of this study have the potential to support the development of sustainable nano-priming and nano-foliar strategies, thereby promoting improved growth performance in rice seedlings subjected to As stress.

## Experimental materials and methods

### Green synthesis of SeNPs and characterization

#### Preparation of raisin (*Vitis vinifera*) extract

High table quality raisins of Happilo brand were obtained from the market and soaked overnight in distilled water. The raisins were blot-dried to remove excess water and uniformly macerated in a blender to make a fine slurry. The obtained slurry was filtered three times with Whatman filter paper no. 42 until a clear filtrate was obtained. After being refluxed for 40 min at 70 °C, the filtrate was let to cool at the room temperature. After reflux, the extract was stored, for later use at −4 °C^32^.

#### Preparation of SeNPs

SeNPs of the ideal size were synthesised using a range of sodium selenite concentrations, ranging from 10 mM to 30 mM^32^. The resulting raisin extract was mixed with the sodium selenite solution at a 1:9 ratio and refluxed for 30 minutes at 90 °C in the reflux unit. After that the color of the reaction mixture changed from pale yellow to red and finally dark brick-red which indicates the synthesis of SeNPs. The reflux mixture was allowed to cool and then collected in vials. The SeNPs solution was washed alternatively with ethanol and distilled water 15 times and the last wash was performed with distilled water. The washes were performed via a centrifuge (Eppendorf 5418R, Eppendorf, Hamburg, Germany) 15 min at 14,000 rpm each time. Furthermore, the washed SeNP solution was sonicated for 45 min (25 kHz, 250 W, LABMAN).The sonicated SeNPs were dried in desiccators and desiccated SeNP was further allowed to dry in a lyophilizer. For characterization along with efficiency tests, the lyophilized SeNPs were ground into a powder and kept in an airtight container at 20 °C^32^.

#### Characterization of green synthesized SeNPs

Preliminary characterization was performed via a UV-visible spectrophotometer (Labtronics LT-2201, India) and absorbance was recorded from 190 nm to 1100 nm using the wavelength scan function. The size and shape of the SeNP particles were determined via field emission scanning electron microscopy (FE-SEM) analysis (EVO SEM MA 15/18, Carl-Zeiss, Germany). High-resolution transmission electron microscopy (TEM) was used to characterize the morphology and microstructures of the biogenic SeNPs (HRTEM, JEOL JEM-1230, EDAX Inc., USA). FE-SEM with EDS analysis was used to characterize the elemental composition of the particles.

### Plant materials and growth conditions

Seeds of the rice genotype HUR-105 were sourced from the Department of Genetics and Plant Breeding, Institute of Agricultural Sciences, Banaras Hindu University (BHU), Varanasi. The experiment was conducted at the Department of Plant Physiology, BHU, located at 25°15’ N, 82°59’ E, and 75.7 m above sea level. Uniform, mature seeds were cleaned, soaked in distilled water for 24 h, and surface sterilized with 1% NaOCl for 10 min. Sodium arsenite (100 µM) was used as the As source. The control (Se0+As0) was maintained in Hoagland solution without As or Se Hoagland and Arnon Hoagland and Arnon^41^. Sodium selenite (Se) and SeNPs were applied at 10 µM and 20 µM (Se), and 20 µM and 25 µM (SeNPs), labeled Se10, Se20, SeNP20, and SeNP25 respectively. Treatments were applied through three modes: seed priming, co-application in solution, and foliar spray. Each combination, including Se+As treatments (Se10+As, Se20+As, SeNP20+As, and SeNP25+As), was tested in all modes with four replicates per treatment.

To assess growth, morphology, and biochemical responses, rice seedlings were initially grown for 5 days on Petri plates supplemented with half-strength modified Hoagland solution. Uniform seedlings were then transferred to PVC cups (12 cm × 11 cm, 40 seedlings per cup per treatment) containing coco peat and irrigated with full-strength Hoagland solution. After 3 days, at the V2 growth stage, the seedlings were treated with the specified As and Se combinations. The plants were maintained in a controlled growth chamber (Caltan-193, NSW, New Delhi) under a 14/10 h light/dark cycle, 400 μmol m⁻^2^ s⁻^1^ PAR, 35 °C/28 °C day/night temperatures, and 70% relative humidity. The nutrient solution was adjusted to pH 5.8. Morpho-physiological parameters- including shoot length, root volume, and leaf count were recorded at 10-day intervals up to 30 days after germination. Biochemical parameters were assessed 10 days after treatment.

### Analytical methods

#### Determination of seedling growth indicators

Seeds were considered germinated when both the plumule and radicle extended from their junction and extended up to 2 mm. Using a measuring scale, the shoot length (SL) of the seedlings was determined. The root length (RL) and root volume (RV) were measured via an Epson Biovis Root scanner at every 10 days till 30 days. The leaf number and leaf area were recorded 10, 20 and 30 days after germination.

#### Determination of seedling biochemical parameters

The total chlorophyll content was estimated in freshly harvested leaves at 30 DAG via the method described by Hiscox and Israelstam Hiscox and Israelstam^42^. The anthrone reagent was used to quantify the quantity of total soluble sugars and starch in accordance with the methods described by Dubois et al. Dubois et al.^43^ for sugars and Hodge and Hofreiter Hodge and Hofreiter^44^ for starch. The method developed by Bradford Bradford^45^ was followed for estimation of total soluble protein. Total free amino acids were determined in fresh rice leaf samples via the use of ninhydrin reagent^46^.

#### Determination of oxidative stress markers

The chlorophyll stability index (CSI) was calculated following the method provided by Kaloyereas Kaloyereas^47^ and the membrane stability index (MSI) was computed via the methodology presented by Azimi et al. Azimi et al.^48^.

#### Histochemical localization in shoots and roots

The accumulation of the free radicals H_2_O_2_ and O_2_^-^ was assessed via 3,3’diaminobenzidine (DAB), and nitro-blue tetrazolium (NBT) respectively via the method described by Awasthi et al. Awasthi et al.^15^ in leaves and roots at 20 DAS and 5 days after treatment by manually removing each leaf and root from the plant and placing them in 2 ml microcentrifuging tubes (MCTs) and all the samples were photographed with a SteREOLumarV12 stereomicroscope.

### Statistical analysis

The data presented are the averages of three replicates. All the data collected were analyzed by R-studios software. The data were subjected to one-way analysis of variance at the 0.01 level of significance. Where significant, the means were compared at the 1% significance level (α = 0.01). Graphs were plotted via OriginPro 2024 and 2025 software with error bars depicting the standard error of the mean.

## Results

### Characterization of green synthesized SeNPs

**Fig. 1:**
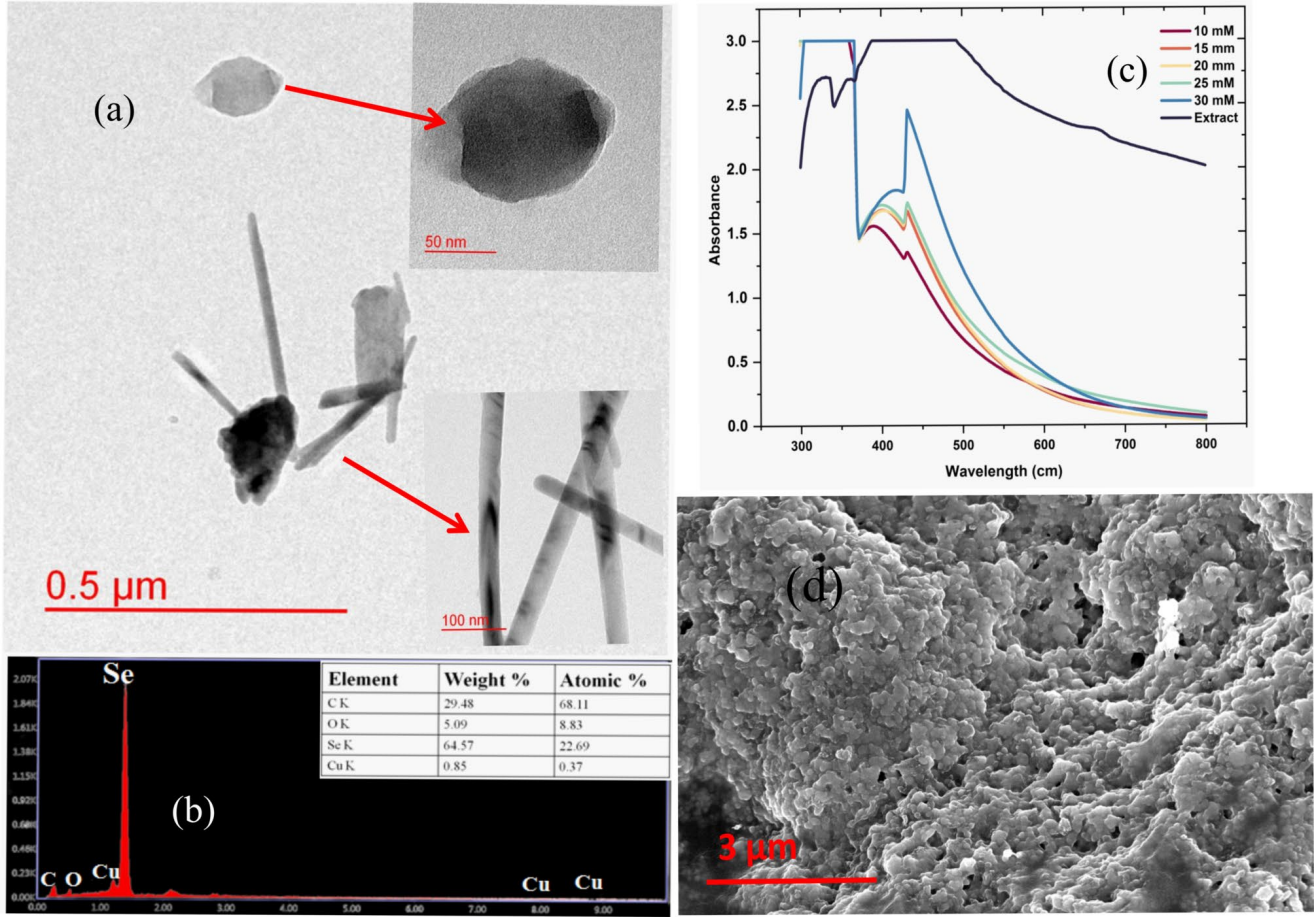
Characterization of green synthesized SeNP (**a**) TEM image of SeNP at 0.5 μm, 50 nm and 100 nm (**b**) EDX spectrum and elemental composition of SeNP (**c**) UV–visible spectrum (**d**) SEM image of SeNP at 3 μm.

SeNPs were synthesized via a green synthesis method by refluxing sodium selenite with *Vitis vinifera* raisin extract. A visible color shift from pale yellow to brick red indicated successful SeNP formation. During the reaction, Se ions from sodium selenite were reduced by phytochemicals in the raisin extract. UV–visible spectroscopy confirmed SeNP synthesis, with a characteristic absorption peak at 430 nm, within the 400–450 nm range. The absorption peak at wavelengths between 300 and 500 nm is shown in Fig. 1.c. Lower concentrations (10 mM) produced smaller particles, as indicated by a blue shift, but with a lower yield. An optimal concentration of 25 mM yielded stable SeNPs, which were used for further analysis. At 30 mM, particle aggregation occurred, as indicated by a secondary peak near 500 nm and increased absorbance in the red region.

SEM analysis (Fig. 1d) revealed that SeNPs synthesized from *Vitis vinifera* raisin extract had smooth surfaces with oval to quasi-spherical shapes and were 90–110 nm in diameter. ImageJ software was used for size determination, confirming the production of stable SeNPs. The elemental composition was confirmed through FE-SEM-EDX and point EDX analyses, which revealed strong peaks at 1.35 kV and the presence of Se (64.57%), carbon (29.48%), oxygen (5.09%), and copper (0.85%) (Fig.1.b). TEM images (Fig. 1.a) revealed spherical SeNPs and actinomorphic Se nanorods (trigonal Se), with particle sizes ranging from 50 to 100 nm and an average diameter of 85 nm.

### Seedling growth parameters

**Fig. 2:**
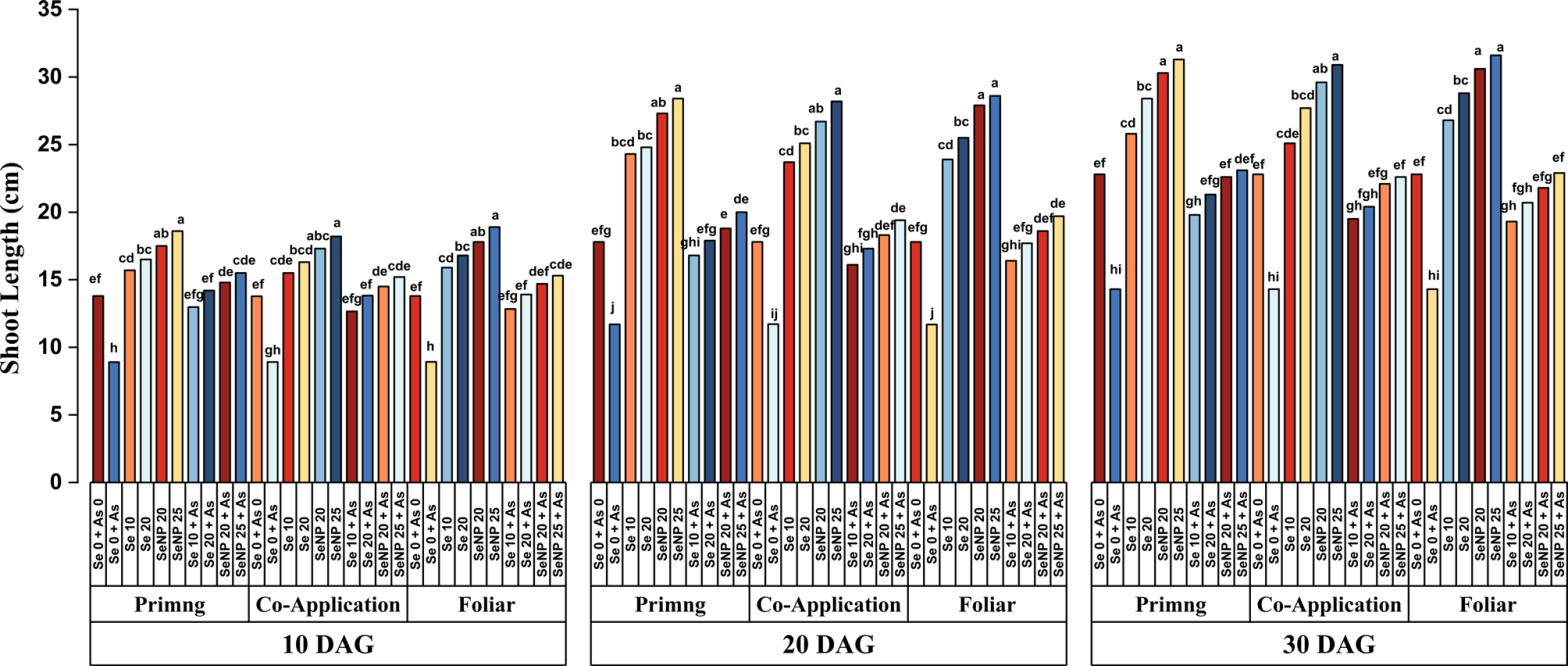
Effect of Selenite salt (Se) and SeNP applied with three different modes (Priming, Co-application and Foliar) on Shoot length (cm)in rice under arsenic stress at (**a**) 10, (**b**) 20 and (**c**) 30 days after germination. All values are the mean of four replicates (±SD). ANOVA significant at (p < 0.01). Different letters indicate significantly different values in a particular tissue (p < 0.01).

As shown in Fig. 2, shoot length varied significantly across treatments at 10, 20, and 30 days after germination (DAG). Arsenic exposure resulted in a pronounced reduction in shoot length, particularly during early growth, with a 45% decrease observed at 10 DAG (Fig. 2.a). In contrast, the Se treatments, especially SeNP 25 applied via foliar spray, positively influenced shoot growth at all stages. Shoot length progressively increased over time, reaching a maximum of 31.60 cm at 30 DAG (Fig. 2.c). However, the most substantial increase relative to the control (60%) occurred at 20 DAG (Fig. 2.b). Among all the treatments, SeNP 25 + As in the priming mode demonstrated the most effective mitigation of As-induced growth inhibition at all the time points, followed closely by the same treatment applied via the foliar mode.

**Fig. 3:**
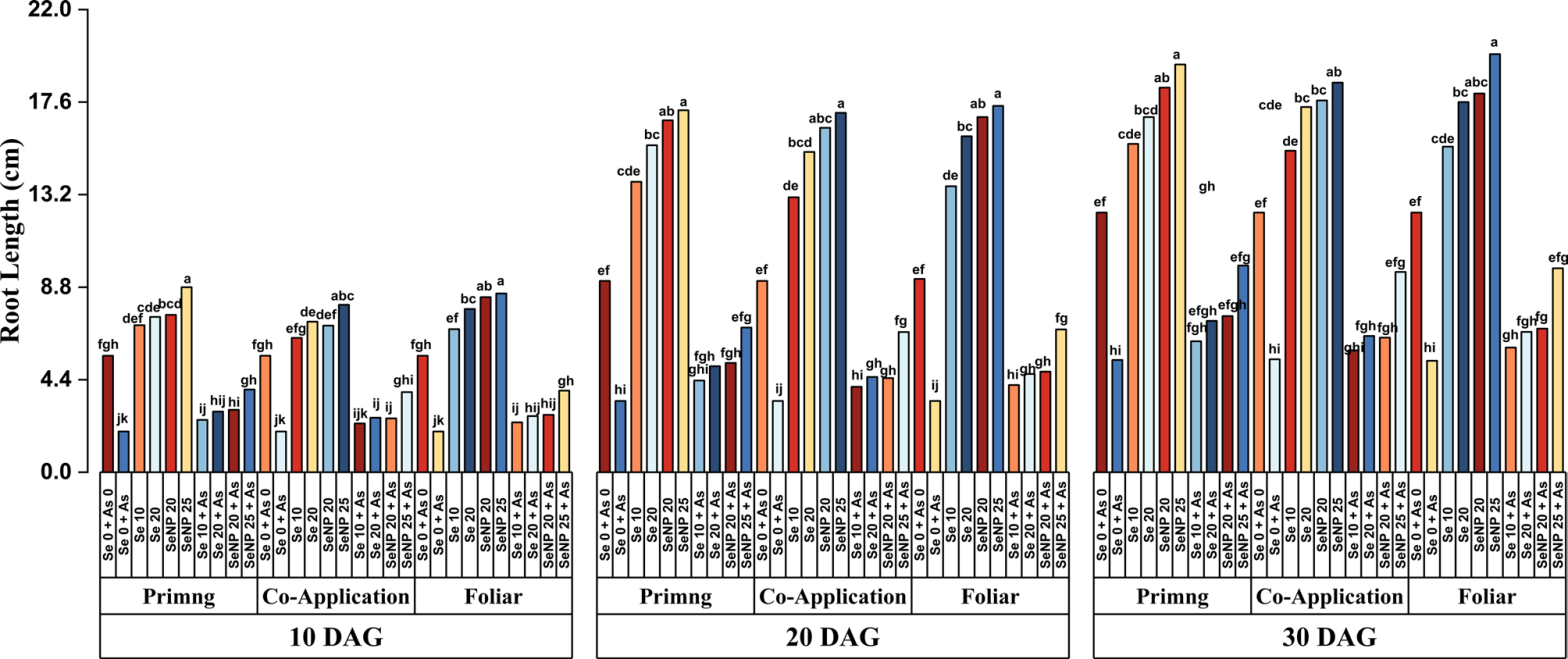
Effect of Selenite salt (Se) and SeNP applied with three different modes (Priming, Co-application and Foliar) on Root length (cm)in rice under arsenic stress at (**a**) 10, (**b**) 20 and (**c**) 30 days after germination. All values are the mean of four replicates (±SD). ANOVA significant at (p < 0.01). Different letters indicate significantly different values in a particular tissue (p < 0.01).

**Fig. 4:**
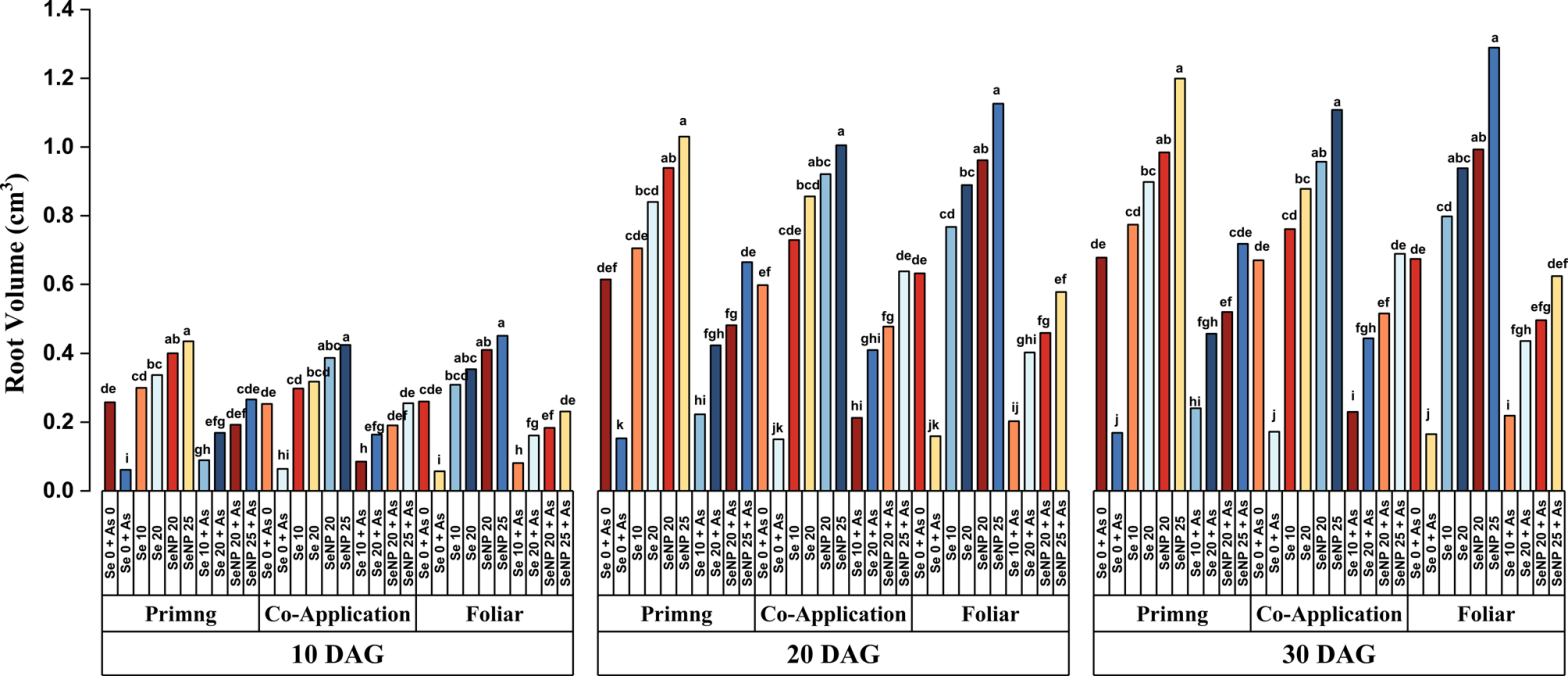
Effect of Selenite salt (Se) and SeNP applied with three different modes (Priming, Co-application and Foliar) on Root volume (cm^3^)in rice under arsenic stress at (**a**) 10, (**b**) 20 and (**c**) 30 days after germination. All values are the mean of four replicates (±SD). ANOVA significant at (p < 0.01). Different letters indicate significantly different values in a particular tissue (p < 0.01).

Figures 3 and 4 show that As suppressed root development more severely than shoot growth. Compared with those in the untreated control (Se0 + A0), the root length decreased by more than 70%, and the root volume decreased by more than 90%, with the steepest decline (90% in root length) occurring at 10 DAG (Fig. 3a). On the other hand, Se counteracted this inhibition. SeNP 25, particularly via foliar application, consistently enhanced root growth. The root length reached 17.42 cm at 20 DAG, an 89% increase over the control (Fig. 3b), whereas the root volume peaked at 1.29 cm^3^, a 91% increase, at 30 DAG (Fig. 4c). Across application modes, SeNP 25 + As delivered as a seed priming agent provided the most effective mitigation at every time point, followed closely by the same formulation applied foliarly.

As shown in Figures 2,3,4 seed priming with 25 µM SeNPs significantly enhanced seedling growth under As stress. Arsenic exposure led to a marked reduction in leaf number and leaf area (Tables 1 and 2), with up to 90% decrease at 10 DAG and 75% decrease at later stages. In contrast, the Se treatments improved leaf growth, particularly in leaf area, with SeNP 25 resulting in the greatest increase of nearly 95% at 10 DAG. Although all the Se application modes were effective, the differences among them were not statistically significant. The leaf number increased over time but showed no major variation across the Se treatments, indicating that the effects of Se salt and SeNPs were comparable. Overall, the SeNP treatments, especially the SeNP 25 + As foliar treatments, had the most consistent and pronounced ameliorative effects, confirming the superior efficacy of SeNPs in promoting seedling growth under As toxicity.

**Table 1:** Effects of three different modes of selenite salt (Se) and SeNPs (priming, coapplication and foliar) on the number of leaves per seedling of rice under arsenic stress at 10, 20 and 30 days after germination.

**Leaf number/seedling**									
**Treatments**	**10 DAG**			**20 DAG**			**30 DAG**		
	**Priming**	**Co-application**	**Foliar**	**Priming**	**Co-application**	**Foliar**	**Priming**	**Co-application**	**Foliar**
**Se 0 + As 0**	2±0.007^ab^	2±0.024^ab^	2±0.044^ab^	3±0.004^bc^	3±0.020^bc^	3±0.041^bc^	5±0.12^ab^	5±0.12^ab^	5±0.02^ab^
**Se 0 + As**	1±0.003^b^	1±0.015^b^	1±0.016^b^	2±0.026^c^	2±0.002^c^	2±0.049^c^	3±0.02^c^	3±0.06^c^	3±0.04^c^
**Se 10**	2±0.004^ab^	3±0.062^a^	3±0.028^a^	3±0.055^bc^	4±0.100^ab^	4±0.004^ab^	5±0.04^ab^	6±0.05^a^	6±0.14^a^
**Se 20**	3±0.020^a^	3±0.037^a^	3±0.017^a^	4±0.042^ab^	4±0.010^ab^	4±0.008^ab^	6±0.06^a^	6±0.04^a^	6±0.00^a^
**SeNP 20**	3±0.003^a^	3±0.069^a^	3±0.017^a^	5±0.062^a^	5±0.057^a^	5±0.122^a^	6±0.02^a^	6±0.01^a^	6±0.02^a^
**SeNP 25**	3±0.077^a^	3±0.061^a^	3±0.077^a^	5±0.013^a^	5±0.010^a^	5±0.101^a^	6±0.05^a^	6±0.15^a^	6±0.04^a^
**Se 10 + As**	1±0.024^b^	1±0.022^b^	2±0.003^ab^	3±0.069^bc^	2±0.009^c^	3±0.014^bc^	4±0.09^bc^	4±0.05^bc^	5±0.09^ab^
**Se 20 + As**	2±0.018^ab^	1±0.019^b^	2±0.005^ab^	3±0.028^bc^	3±0.041^bc^	3±0.077^bc^	5±0.00^ab^	5±0.03^ab^	5±0.05^ab^
**SeNP 20 + As**	2±0.006^ab^	2±0.034^ab^	3±0.062^a^	4±0.090^**ab**^	3±0.061^bc^	4±0.100^ab^	5±0.03^ab^	5±0.10^ab^	6±0.10^a^
**SeNP 25 + As**	2±0.017^ab^	3±0.061^a^	3±0.028^a^	4±0.069^ab^	5±0.003^a^	5±0.109^a^	5±0.09^ab^	6±0.06^a^	6±0.03^a^
**LSD**									
**Factors**	**M**	**C**	**M** x** C**	**M**	**C**	**M** x** C**	**M**	**C**	**M** x** C**
**SE(d)**	0.017	0.031	0.053	0.026	0.048	0.083	0.031	0.057	0.099
**CD (1%)**	0.044	0.081	0.141	0.070	0.128	0.222	0.083	0.152	0.264

^*^Duncan’s multiple range test (DMRT) post-hoc test was performed at α = 0.01. The mean in each column followed by the same letter are not significantly different.

**Table 2:** Effects of three different modes of selenite salt (Se) and SeNPs (priming, coapplication and foliar) on the leaf area (cm^2^) of rice under arsenic stress at 10, 20 and 30 days after germination.

Leaf Area (cm^2^)																
Treatments		10 DAG					20 DAG						30 DAG			
		Priming	Co-application		Foliar		Priming		Co-application			Foliar	Priming	Co-application		Foliar
Se 0 + As 0		16.94±0.44^ef^	16.29±0.32^ef^		16.64±0.01^ef^		27.98±0.36^e^		27.66±0.45^e^			27.49±0.48^e^	40.41±0.33^def^	40.78±0.42^def^		40.29±0.96^def^
Se 0 + As		9.13±0.03^gh^	9.51±0.17^gh^		9.29±0.21^gh^		10.57±0.10^i^		10.74±0.03^i^			10.38±0.02^i^	15.00±1.30^i^	15.58±0.15^i^		15.29±0.34^i^
Se 10		24.87±0.82^cd^	23.69±0.09^cd^		24.26±0.27^cd^		43.21±1.03^bc^		42.83±0.51^bcd^			43.67±1.05^bc^	65.36±1.53^bc^	63.27±0.27^bcd^		64.58±0.17^bcd^
Se 20		26.04±0.06^bc^	25.76±0.35^bcd^		26.48±0.32^bc^		47.88±1.00^abc^		46.94±0.93^ab^			47.26±0.51^abc^	66.34±0.52^abc^	65.78±1.72^bc^		66.78±1.42^abc^
SeNP 20		29.72±0.79^ab^	28.65±0.99^b^		30.08±0.60^ab^		51.21±0.91^a^		48.85±0.99^abc^			49.73±0.23^ab^	68.18±0.64^ab^	68.67±0.37^ab^		69.37±1.26^ab^
SeNP 25		31.97±0.98^a^	32.56±0.02^a^		32.83±0.42^a^		52.11±0.79^a^		52.76±0.19^a^			53.58±0.22^a^	70.57±0.29^a^	71.68±0.37^a^		72.89±1.18^a^
Se 10 + As		15.41±0.24^fg^	15.06±0.19^fg^		15.88±0.26^fg^		18.97±0.09^hi^		18.03±0.14^hi^			19.73±0.09^ghi^	32.78±0.34^gh^	32.19±0.65^gh^		34.89±0.87^fgh^
Se 20 + As		16.19±0.02^fg^	15.81±0.38^fg^		16.34±0.25^fg^		21.28±0.44f^gh^		21.01±0.02^fgh^			22.67±0.42^efgh^	35.63±0.22^fgh^	35.41±0.17^fgh^		36.87±0.40^efg^
SeNP 20 + As		16.83±0.43^efg^	16.48±0.09^efg^		17.37±0.18^fg^		24.15±0.01^ef^		23.82±0.24^efg^			24.67±0.59^ef^	39.84±0.52^def^	39.24±0.12^ef^		40.98±0.92^def^
SeNP 25 + As		18.86±0.20^def^	19.98±0.02^de^		21.23±0.46^cde^		25.99±0.42^e^		25.49±0.56^e^			27.58±0.1^3e^	42.89±0.58^cde^	42.11±0.53^de^		44.16±0.46^cde^
**LSD**																
**Factors**	**M**			**C**		**M x C**		**M**		**C**	**M x C**		**M**	**C**	**M x C**	
SE(d)	0.19			0.34		0.59		0.25		0.45	0.78		0.33	0.60	1.04	
CD (1%)	0.50			0.91		1.58		0.65		1.19	2.07		0.88	1.60	2.78	

^*^Duncan’s multiple range test (DMRT) post-hoc test was performed at α = 0.01. The mean in each column followed by the same letter are not significantly different.

### Biochemical parameters

**Fig. 5:**
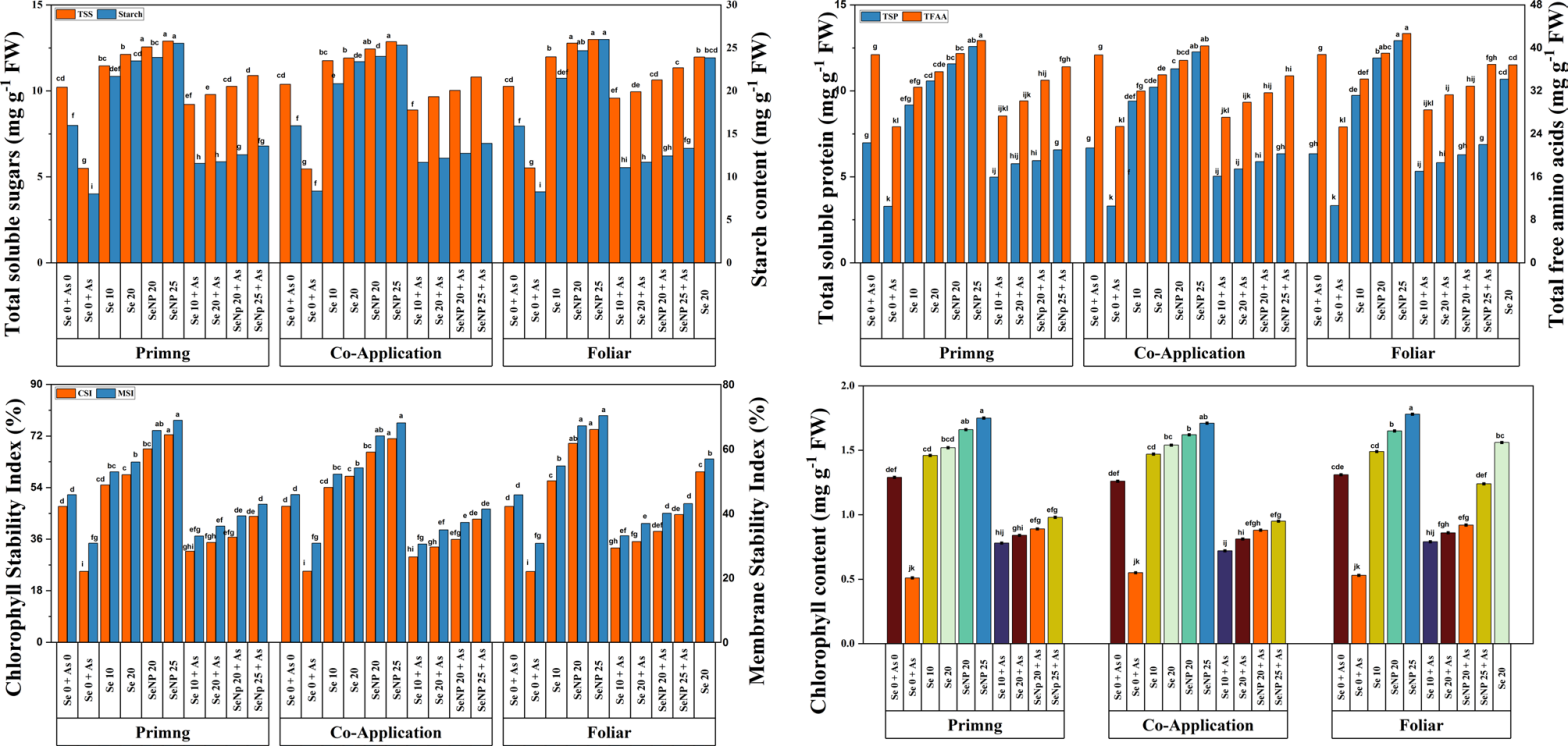
Effect of Selenite salt (Se) and SeNP applied with three different modes (Priming, Co-application and Foliar) on, (**a**) Total Soluble Sugar content (mg g^−1^ FW) and Starch content (mg g^−1^ FW), (**b**) Total Soluble Protein content (mg g^−1^ FW) and Total Free Amino Acids content (mg g^−1^ FW), (**c**) Chlorophyll Stability Index and Membrane Stability Index, and (**d**) Total Chlorophyll content (mg g^−1^ FW)in the leaves of rice under arsenic stress. All values are the mean of four replicates (±SD). ANOVA significant at (p < 0.01). Different letters indicate significantly different values in a particular tissue (p < 0.01).

As shown in Fig. 5, As stress significantly impaired key biochemical parameters in the rice seedlings. The chlorophyll content declined by approximately 70% compared with that of the control (Se0 + A0) (Fig. 5d), whereas the total soluble sugar and starch contents decreased by more than 50% and 55%, respectively (Fig. 5a). Similarly, the total protein and free amino acid levels contents decreased by approximately 55% and 35%, respectively (Fig. 5b). In contrast, Se application notably improved the biochemical response. Compared with the control foliar application of SeNP 25 under As stress resulted in the greatest increase in total chlorophyll (1.78 mg g⁻^1^ FW), soluble sugar (12.44 mg g⁻^1^ FW), starch (25.98 mg g⁻^1^ FW), protein (12.92 mg g⁻^1^ FW), and free amino acid (42.69 mg g⁻^1^ FW) contents, corresponding to increases of 38%, 27%, 63%, 71%, and 10%, respectively. All the Se application modes (priming, co-application, and foliar) demonstrated ameliorative effects under As stress, with SeNP 25 + As foliar mode resulting in the most significant improvement across all the parameters- except the starch content, which was highest under co-application. Overall, SeNP treatments were more effective than Se salt treatments in enhancing biochemical parameters and mitigating As toxicity in rice seedlings.

### Determination of oxidative stress markers

As shown in Fig. 5.c, As stress significantly reduced the CSI and MSI in rice leaves by approximately 46% and 28%, respectively, compared with those of the control (Se0 + A0). In contrast, Se application improved both indices, indicating enhanced stress tolerance. Among all treatments, SeNP 25 applied via the foliar mode resulted in the greatest increase- 67% in the CSI and 73% in the MSI over the control with absolute values of 44.56 and 42.99, respectively. All modes of Se application (priming, co-application, and foliar) demonstrated ameliorative effects under As stress, with SeNP treatments consistently outperforming Se salt. Overall, foliar application of SeNP 25 was most effective at increasing the stability of the membrane and chlorophyll under As toxicity in rice seedlings.

### Histochemical staining detection of ROS (O_2_^–^ and H_2_O_2_)

**Fig. 6:**
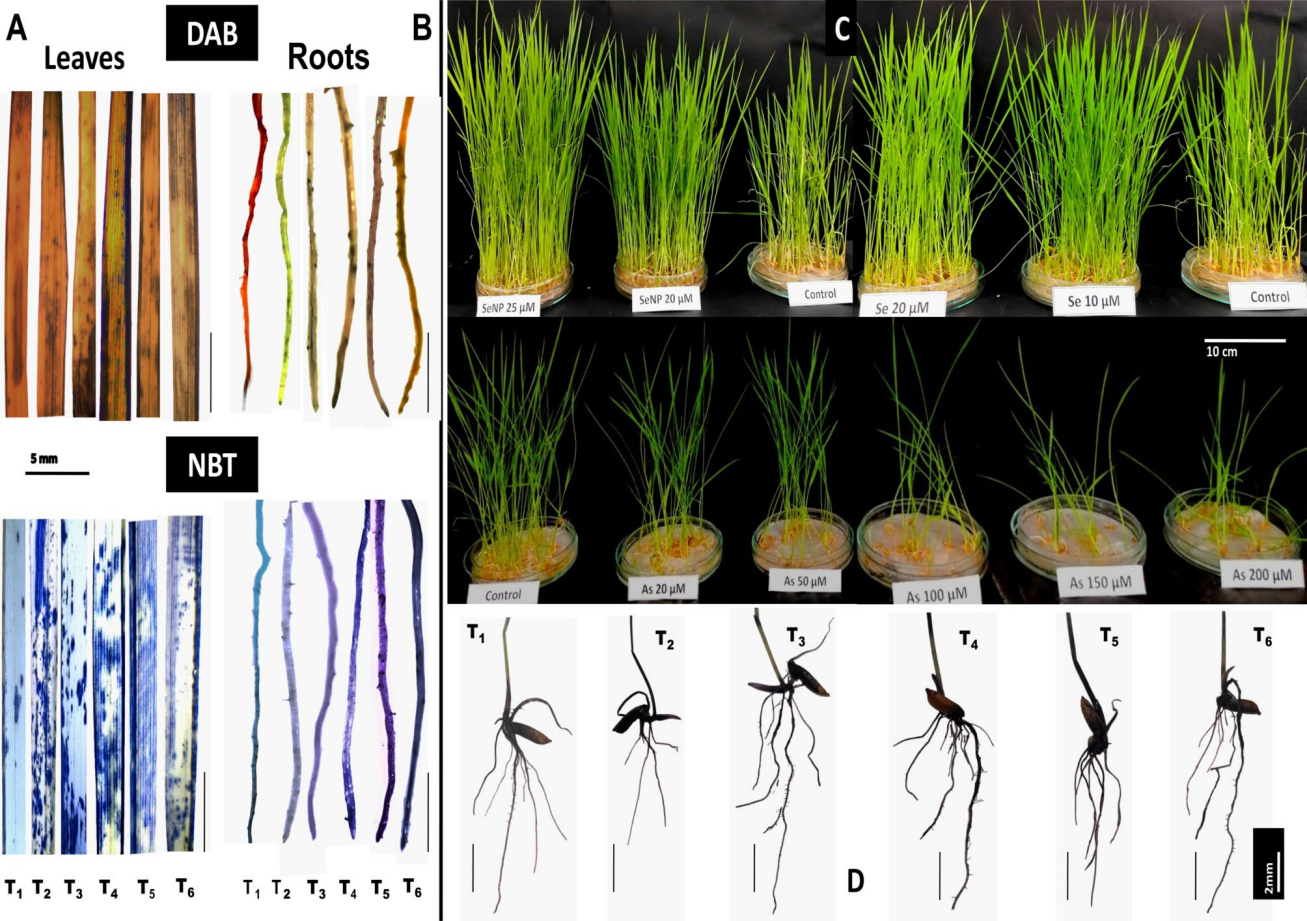
Histochemical detection (**A**) H_2_O_2_ in leaves and roots stained with DAB (Upper side), (**B**) O_2_^−^ in leaves and roots stained with NBT (Lower side) (Scale bar = 5 mm), (**C**) 12 days old seedlings after 7 days of treatment(Scale bar = 10 cm), and (**D**) Roots of seedlings after 7 days of treatment(Scale bar = 2 mm). (T_1_ (Control), T_2_ (100 µM arsenite) T_3_ (20 µM selenite salt), T_4_ (25 µM SeNP), T_5_ (As 100 µM +20 µM selenite salt), and (As 100 µM +25 µM SeNP).

As depicted in Fig. 6.A and B, DAB and NBT staining revealed intense brown and blue coloration in leaves and roots, respectively, under As treatment, indicating elevated levels of hydrogen peroxide (H₂O₂) and superoxide (O₂⁻), confirming severe oxidative stress compared with that in the control (Se0 + A0) and Se-treated plants (T_3_ and T_4_). In contrast, Se application under As stress (T_5_ and T_6_) significantly reduced ROS accumulation, as evidenced by lighter staining in both tissues than that in the As-only treatment (T_2_). Notably, SeNP application (T_6_) showed the greatest reduction in ROS levels, with minimal brown and blue coloration observed in both root and shoot tissues. These findings indicate that SeNPs effectively mitigated the oxidative stress induced by As exposure.

### Correlation studies

**Fig. 7:**
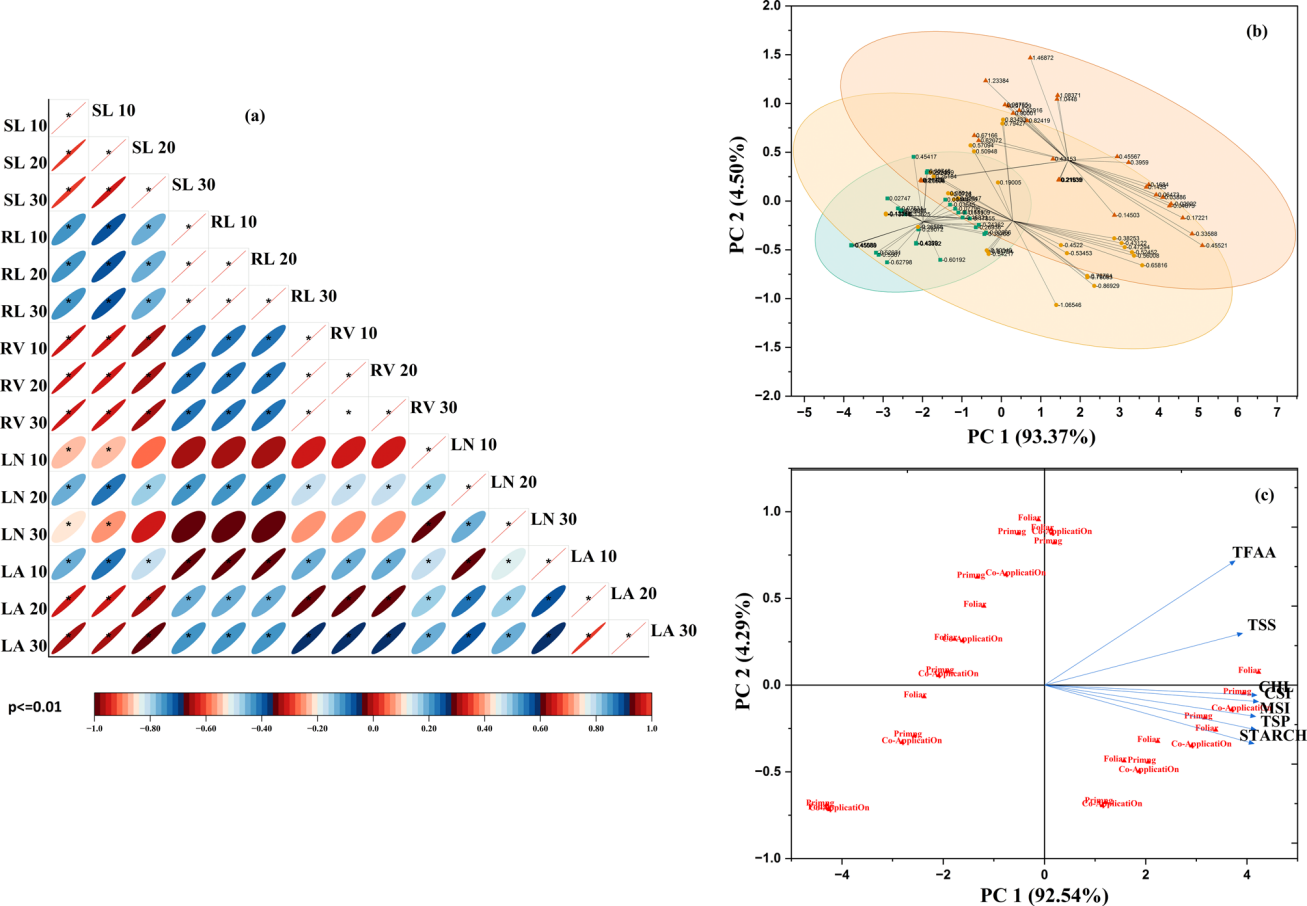
Heatmap of Pearson’s Correlation matrix indicating the responses of morphological traits (**a**), PCA Cluster Biplot (**b**) and Loading Chart of PCA-Biplot (**c**) of biochemical parameters in rice seedlings under arsenic and selenium (Se, SeNP) treatments.

Figure 7.a presents a heatmap of the Pearson correlation matrix for seedling growth parameters (shoot length, root length, root volume, leaf number, and leaf area) at 10, 20, and 30 DAG in rice seedlings treated with Se salt and SeNPs under arsenite stress. All the parameters were significantly positively correlated (p ≤ 0.01) except for leaf number, which was not significantly correlated with the other variables at any stage. Figures 7.b and 7.c display the PCA cluster biplot and loading chart for the biochemical parameters, respectively. The PCA results indicate strong positive correlations among all the biochemical parameters (p ≤ 0.01), with PC_1_ explaining 92–94.3% and PC_2_ accounting for 4.2–4.5% of the variance.

## Discussion

HM toxicity is a serious threat to food production at both the quantitative and qualitative levels endangering food security. The competitive inhibition of the uptake of one heavy metal by another has now been established as a promising strategy. Arsenic has emerged as a major pollutant especially in rice production systems thus, causing yield and quality losses. Hence, in the current study the effects of Se supplied as nanoparticle for the mitigation of As toxicity were studied. Se is an essential micronutrient for humans and animals, and plays key roles in antioxidant defense, cellular metabolism, and the regulation of mineral absorption^49^. Both As and Se are known to accumulate in rice,however, the mechanisms by which Se alleviates As toxicity in rice genotypes are not fully understood. In this study, SeNPs were synthesized via a green synthesis method via *Vitis vinifera* extract, which is known for its therapeutic properties, including antimicrobial, antiviral, anti-inflammatory, antioxidant, anti-glycemic, and anticancer effects^50^. The resulting SeNPs, which are capped with bioactive compounds such as flavonoids, phenolics, and polysaccharides, are stabilized through bioreduction and may serve as effective nanomaterials with potential therapeutic relevance. The morphological features of the SeNPs were examined via SEM, revealing predominantly oval, irregular, and nearly spherical shapes with smooth surfaces (Fig. 1d). Point EDX analysis confirmed the presence of Se along with weak signals for C, O, and Cu, indicating the coating of biomolecules from the raisin extract. These findings are consistent with those of previous studies^34,51,52^. TEM (Fig. 1.a) further revealed the presence of spherical and actinomorphic rod-shaped SeNPs, characteristic of trigonal selenium (t-Se)^52^.The ability of these nanoparticles to mitigate As toxicity was studied in rice seedlings.

Arsenic exposure led to reduced biomass and stunted growth due to its toxic effects on cell division, photosynthesis, membrane integrity, and antioxidant activity^53–55^. In contrast, Se application significantly improved growth and biomass under As stress (Fig. 6.C, D), which is consistent with previous findings depicting its positive role in enhancing plant development and stress tolerance^56^. Previous findings have shown the role of Se in promoting nutrient uptake and utilization^56–58^, which contributes to improved plant growth under stress. Additionally, Se can precipitate with silicon (Si) and other HMs, thus reducing crop heavy metal absorption and transportation^59^ and reducing its negative impacts. The precipitates of Se with silica deposit in cell walls (silicification), forming specific cells (siliceous cells), which improves plant disease and stress resistance^60^. This sequestration also aids in alleviating heavy metal toxicity. One of the major strategies employed by plants to mitigate HM toxicity is to prevent its uptake from the soil or the uptake of alternative less toxic ions. Se application significantly reduces As uptake and accumulation in rice seedlings, particularly in roots^61^. This reduction is attributed to the ability of Se in SeNPs to competitively inhibit As uptake or due to the adsorption of As onto SeNP surfaces, decreasing As mobility and bioavailability in the rhizosphere^62,63^. Additionally, Se enhances the formation of endodermal apoplastic barriers, restricting heavy metal transport into the xylem^64^. The photosynthetic machinery is most susceptible to stress and changes in chlorophyll content are evident quite early in plants experiencing stress. Se treatments significantly increased the total chlorophyll content in rice leaves under As stress, with the highest levels being observed in seedlings treated by foliar spray with 25 μM SeNPs, as observed by Ashraf et al.^65^, Seliem et al.^66^. Stress markedly reduces chlorophyll due to oxidative damage and the inhibition of chlorophyll biosynthesis enzymes^67–69^. This is accomplished by enhancing chlorophyll and carotenoid synthesis, likely through antioxidant action and reorganization of the antenna complex to optimize light capture^70,71^. These effects support improved photosynthesis and growth under As toxicity. HMs toxicity increases the production of reactive intermediates leading to oxidative stress. Se further mitigates heavy metal toxicity by regulating cellular antioxidant systems^25,72^ and facilitating cell repair^73^. This upregulation of the antioxidant machinery helps maintain cellular redox homeostasis thus, reducing the negative impacts of reactive intermediates.

Sugars are essential for plant growth, regulating carbon metabolism and acting as osmoprotectants under stress^74^. In this study, Se treatment significantly increased soluble sugar and starch levels in rice leaves, promoting cell division and overall growth, which is consistent with earlier findings^58,74,75^. In contrast, As stress reduces sugar and starch contents, likely due to disrupted carbohydrate metabolism and impaired water availability^27,76,77^. Notably, Se application under As stress restored sugar and starch levels, with 25 μM SeNP foliar treatment being the most effective at increasing sugar accumulation and maintaining cellular turgor. These findings confirm that Se mitigates As toxicity by promoting sugar accumulation and enhancing starch breakdown, thereby supporting stress tolerance. Sugars serve as vital substrates for sustaining metabolic functions during stress^78^, and the observed increase is likely due to Se-induced activation of amylases^24^ and other carbohydrate-metabolizing enzymes, aiding in osmotic balance and membrane stability^58,76^. Furthermore, Se treatment significantly increased the total soluble protein content in rice seedling leaves, corroborating the findings of Ashraf et al.^65^ and Seliem et al.^66^. Arsenic negatively affects plant growth by inhibiting protein accumulation and increasing oxidative stress, leading to chlorosis and degradation of proteins and amino acids^68,79,80^. Supplementation with selenite and SeNPs mitigated As toxicity by protecting protein structures and enhancing protein synthesis. These results align with prior research linking protein reduction to elevated protease activity and ROS damage^65^.

Cell membrane stability, reflected by MSI and CSI, is essential for stress tolerance in plants^81^. In this study, Se treatment significantly improved the MSI and CSI in rice seedling leaves compared to the control, which is consistent with the findings of Azimi et al. Azimi et al.^48^. Conversely, As exposure reduces MSI and CSI due to membrane lipid peroxidation and increased membrane permeability, which is driven primarily by membrane damage as evidenced by elevated MDA levels^40^. In vivo DAB and NBT staining further confirmed the protective role of Se by indicating lower accumulation of O₂•⁻ and H₂O₂. Se application preserves membrane integrity and reduces lipid peroxidation, thereby enhancing cellular stability^82^. Se mitigates As-induced oxidative stress by limiting As translocation, reducing As mobility in soil, and modifying the cell wall and membranes^83,84^. To combat HM stress, plants activate antioxidant defense systems, including enzymes such as SOD, CAT, APX, GPx, and POD, as well as non-enzymatic antioxidants such as ascorbate, GSH, carotenoids, and tocopherols^58,85,86^. Se also regulates thiol metabolism, restricting As translocation and preserving growth. Elevated GSH levels promote phytochelatins (PC) synthesis, facilitating the detoxification of HMs through the formation of PC-metal complexes, which are sequestered in vacuoles to lower cytosolic metal concentrations^87–89^. SeNPs mitigate As-induced cellular damage by reducing organelle degradation, membrane injury, and NADPH oxidase activity which are primary sources of ROS in plants^58^. Excess ROS lead to the oxidative modification of proteins, such as carbonylation, indicating cellular damage. SeNP treatments have been shown to lower NADPH oxidase, SOD, CAT, APX, and POD activities, thereby limiting ROS generation and protein oxidation. Se also functions as a cofactor for GPx and thioredoxin reductase, enhancing antioxidant defenses. Increased SeNP application increases GPx activity, thereby increasing the oxidative stress tolerance of rice seedlings under heavy metal exposure^90^^,^^58^. The sugar, protein, amino acid, and chlorophyll contents slightly increased across the various Se treatments but significantly increased with the foliar application of 25 μM SeNPs. These findings suggest that while all application methods offer benefits under As stress, foliar treatment with 25 μM SeNPs most effectively mitigates toxicity and enhances physiological and morphological growth. On the basis of improvements in biochemical parameters and stress indicators (CSI and MSI), 25 μM SeNPs was identified as the optimal concentration for mitigating As toxicity stress in rice.

## Conclusion and recommendations

This study demonstrates that foliar application of SeNPs, particularly at 25 μM, effectively alleviates As-induced toxicity in rice seedlings by enhancing antioxidant defense, secondary metabolite synthesis, and chlorophyll preservation. These improvements supported greater photosynthetic efficiency, membrane stability, and accumulation of sugars, starch, and proteins, collectively improving plant growth and physiology under stress. This suggests that SeNPs may serve as an efficient agronomic tool for sustaining rice cultivation in arsenic-affected regions. While the benefits were most pronounced at the seedling stage under a constant As concentration, further research is needed to validate their persistence across different soils, arsenic levels, and growth stages to determine practical dose ranges and long-term benefits. Establishing these parameters will help translate the laboratory findings into reliable field practices, supporting both crop productivity and food safety in arsenic-contaminated areas. From a broader perspective, this work underscores the promise of nanotechnology-based interventions in sustainable agriculture. Future studies should extend these results to field conditions, evaluate the cumulative effects of repeated SeNP applications, and determine the threshold concentrations that balance efficacy with safety. Moreover, exploring the molecular pathways underlying SeNP-mediated tolerance could provide mechanistic insights and inform the development of targeted nano-formulations. Collectively, these findings not only highlight the potential of SeNPs as a practical strategy to improve As tolerance in rice but also lay the groundwork for advancing nanoparticle-based solutions to strengthen crop resilience against multiple abiotic stresses.

## Data Availability

The datasets generated during and/or analyzed during the current study are available from the corresponding author upon reasonable request.

## Abbreviations

CSI: Chlorophyll stability index
MSI: Membrane stability index
DAG: Days after germination
MDA: Malondialdehyde
H_2_O_2_: Hydrogen peroxide
As: Arsenic
Se: Selenium
SeNP: Selenium nanoparticles
ROS: Reactive oxygen species
TEM: Transmission electron microscopy
SEM: Scanning electron microscopy
EDAX: Energy-dispersive X-ray analysis
EDS: Energy-dispersive X-ray spectroscopy
HM: Heavy metals
